# Supplementary material for: Clostridium butyricum improves cognitive dysfunction in ICV-STZ-induced Alzheimer’s disease mice via suppressing TLR4 signaling pathway through the gut-brain axis
Source: PLoS One. 2023 Jun 2;18(6):e0286086. doi: 10.1371/journal.pone.0286086 (PMC10237464; doi:10.1371/journal.pone.0286086)
Supplement: S1 Raw images — (PDF) [file pone.0286086.s001.pdf]

Figure 3. IF staining and Western-blot of p^{Ser404}-tau protein in mouse hippocampal tissues

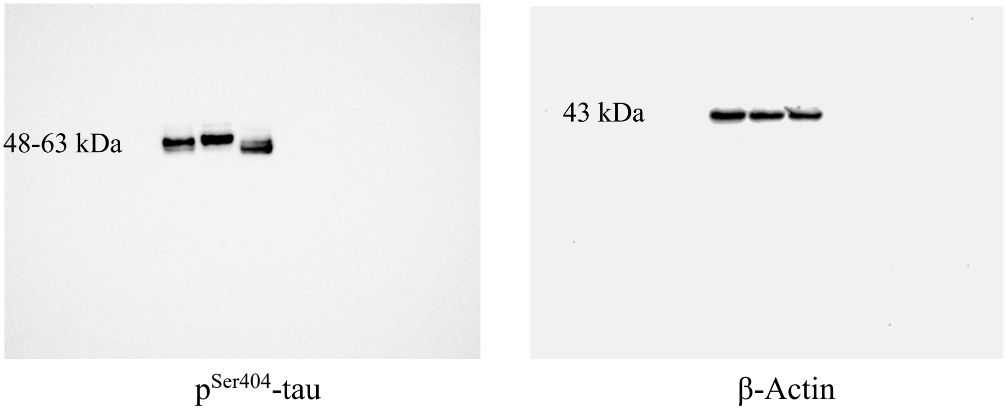

Figure 4. Expression levels of TLR-4, MYD88, NF-κB P65, TNF-α and iNOS in hippocampal tissues of mice in each group

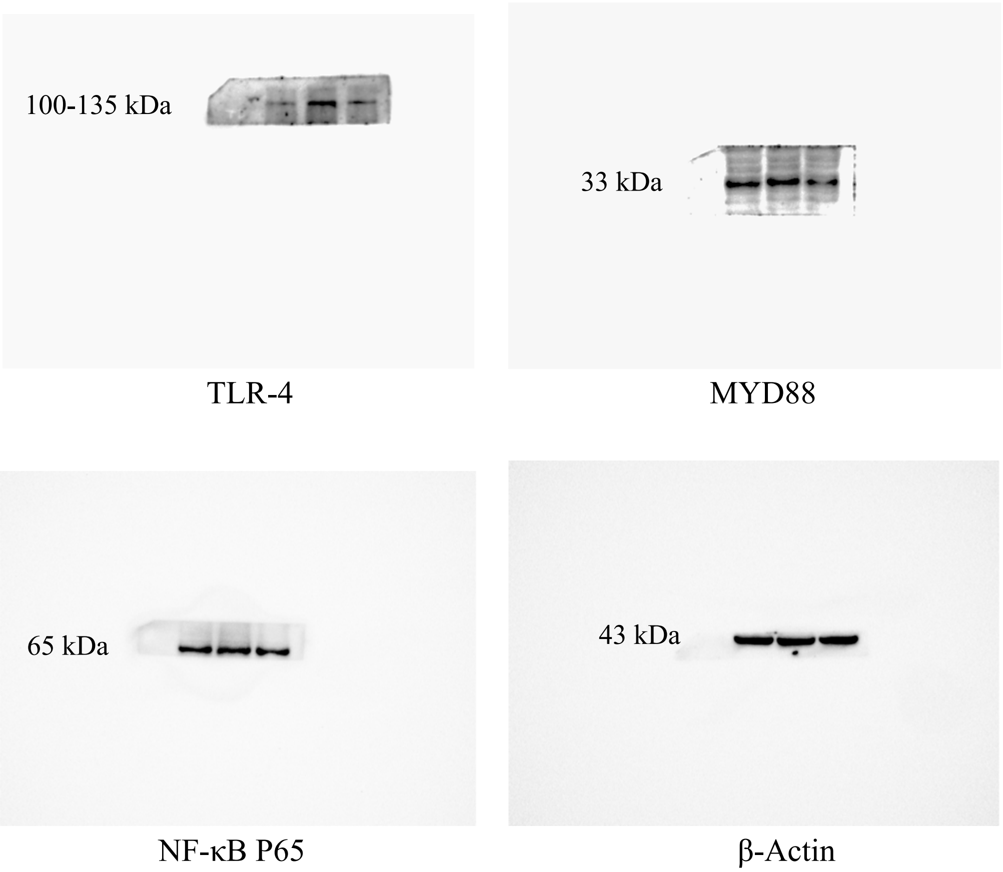

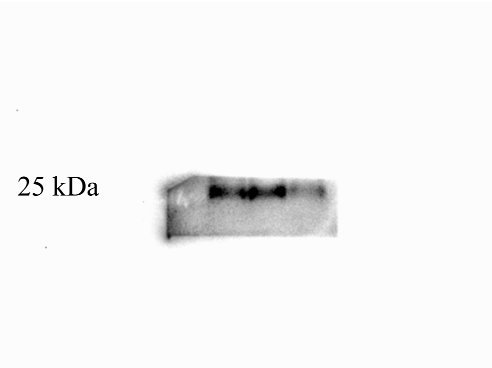

TNF-α

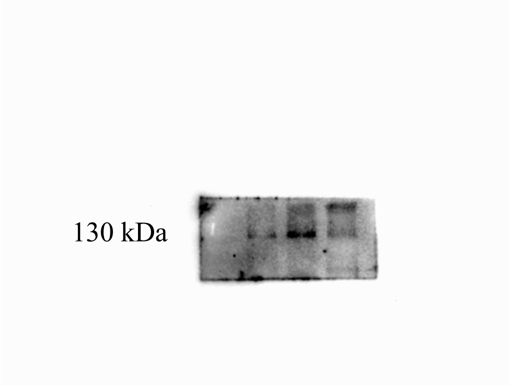

iNOS

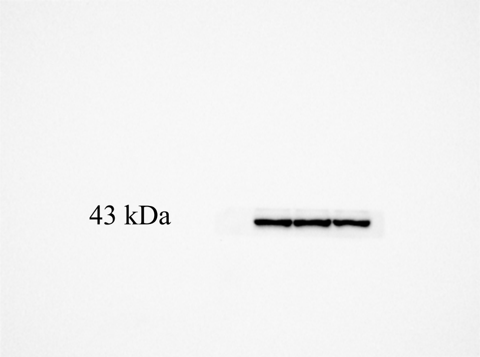

β-Actin

Figure 5. Expression levels of TLR-4, MYD88, NF-κB P65, TNF-α, iNOS, Occludin and ZO-1 in colonic tissues of mice in each group

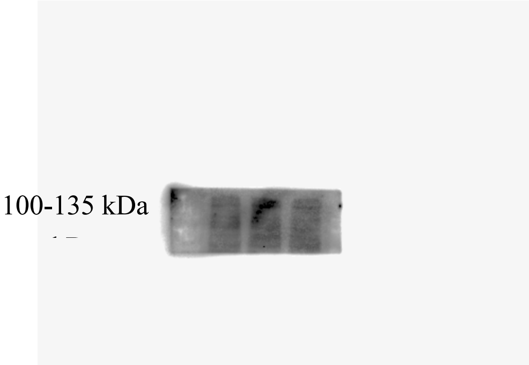

TLR-4

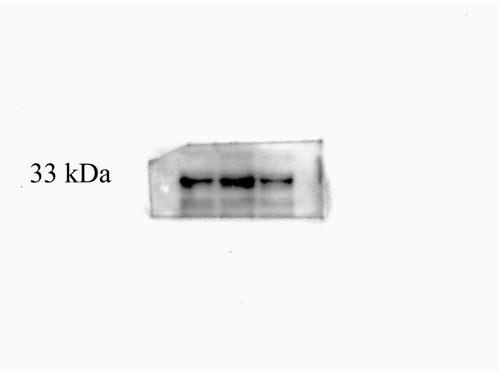

MYD88

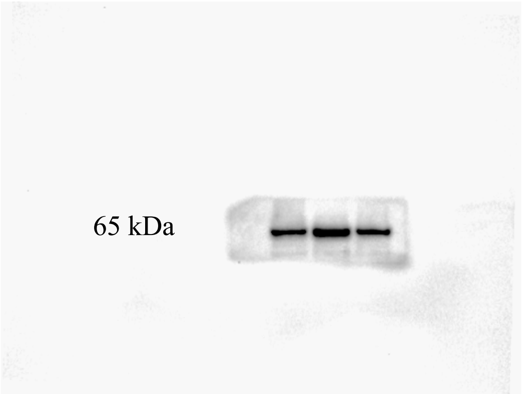

NF-κB P65

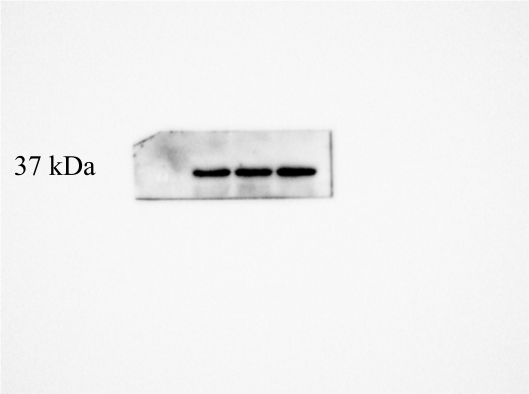

GAPDH

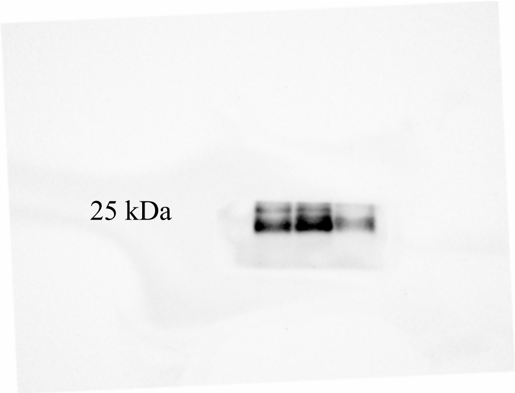

TNF-α

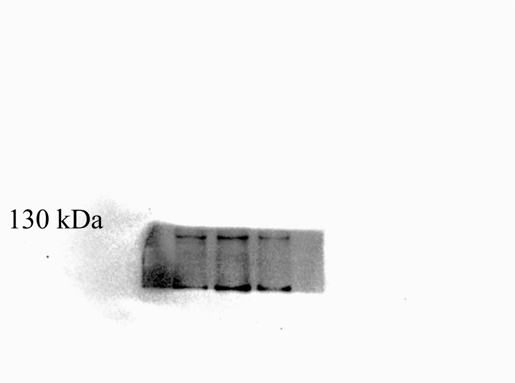

iNOS

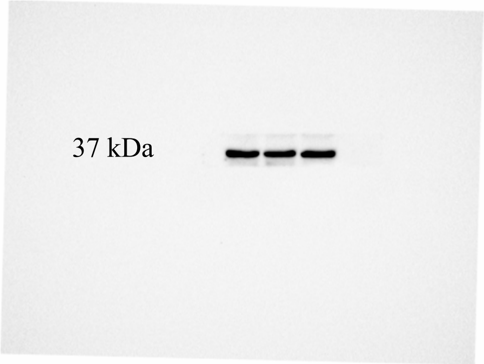

GAPDH

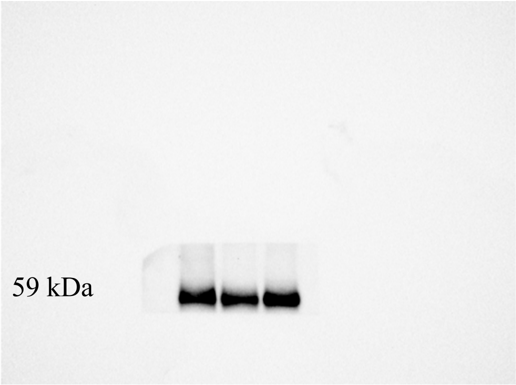

Occludin

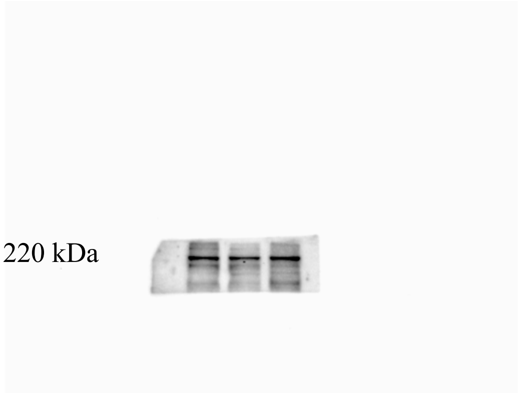

ZO-1

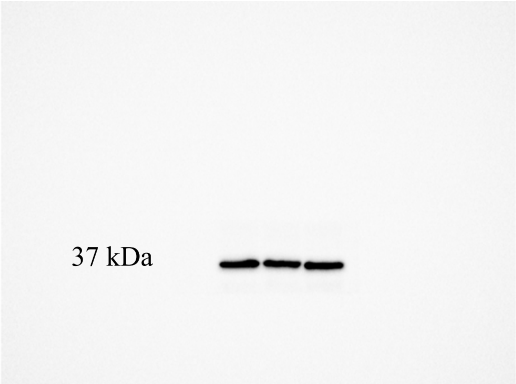

GAPDH
