# Supplementary material for: Clostridium butyricum improves cognitive dysfunction in ICV-STZ-induced Alzheimer’s disease mice via suppressing TLR4 signaling pathway through the gut-brain axis
Source: PLoS One. 2023 Jun 2;18(6):e0286086. doi: 10.1371/journal.pone.0286086 (PMC10237464; doi:10.1371/journal.pone.0286086)
Supplement: Attachment — Submitted filename: Response to Reviewers.docx [file pone.0286086.s005.docx]

**Response to Reviewers**

Dear editors and reviewers:

The authors are grateful to the editors and reviewers for the comments and opportunity to revise the manuscript. The authors responded carefully to the comments and the manuscript was revised. Please give another opportunity to revise again if the editors and reviewers have different opinions or suggestions. Thank you.

Here are the comments and responses:

Reviewer #1: The manuscript entitled ‘Clostridium butyricum improves cognitive dysfunction in ICV-STZ-induced Alzheimer's disease mice via suppressing TLR4 signaling pathway through the gut-brain axis’ is fairly organized, well-written and the results are very interesting. The manuscript just needs English editing with native speaker.

**Response: We agree with this suggestion.** **The manuscript was edited by an English native speaker.**

Reviewer #2: I thank the authors for their tedious work and like to recommend the use of levodopa in addition to pre/probiotics in a third comparison group to study the interaction of both treatment modalities. Thank you.

**Response: Thank you for your suggestion. This study will be better explained if there is a third comparison group to study the interaction of both treatment modalities. In the future, a comparison group with both levodopa and pre/probiotics will be added and the interaction of them will be tested.**

Reviewer #3: "Clostridium butyricum improves cognitive dysfunction in ICV-STZ-induced Alzheimer's disease mice via suppressing TLR4 signaling pathway through the gut-brain axis"

The topic is interesting and study design is sound and well-conducted. The manuscript is organized and well-written, and may be suitable for publication after minor changes.

It is suggested to add a list of abbreviation to help understanding of the lots of abbreviated terms in the text.

Also, the authors, in page 4, lines 106 & 107, stated that "The right ventricle was slowly injected with STZ

107 (3 mg·kg-1) for 5 min", but in many other parts they stated that injected STZ in the lateral ventricle. please revise this sentence.

**Response: Thank you for your suggestion. (1) A list of abbreviation was added in the manuscript (Please see Table 1). (2) The sentence of "The right ventricle was slowly injected with STZ (3 mg·kg-1) for 5 min" was revised to “The right lateral ventricle was slowly injected with STZ (3 mg·kg-1) for 5 min”. Please see the revised manuscript. (lines 110 in the manuscript)**

Sincerely Yours,

Zhenqiang Zhang

Henan Engineering Research Center for Prevention and Treatment of Major Chronic Diseases with Chinese Medicine

Academy of Chinese Medical Sciences

Henan University of Chinese Medicine

156 Jinshui Dong Road

450046 Zhengzhou

Henan province, PR China

Email: [13333719963@126.com](mailto:13333719963@126.com)

Tel: 13333719963

Date: 26-Apirl-2023
